# An Internet-Delivered Intervention to Reduce LGBTQ+ Prejudice Among Romanian Teachers: Randomized Controlled Trial

**DOI:** 10.2196/63787

**Published:** 2026-01-16

**Authors:** Nastasia Sălăgean, Ioana Maria Latu, Torill Marie Bogsnes Larsen, Andreea Bogdana Isbășoiu, Florin Alin Sava

**Affiliations:** 1 Department of Scientific Research in Economy, Law and Human-Environment Interaction, Institute for Advanced Environmental Research, West University of Timișoara, Boulevard V Pârvan 4, Timisoara, 300223, Romania, 40 0256-592-309; 2 Department of Psychology, Faculty of Sociology and Psychology, West University of Timisoara, Timisoara, Romania; 3 School of Psychology, Queen's University Beflast, Beflast, United Kingdom; 4 Department of Health Promotion and Development, Faculty of Psychology, University of Bergen, Bergen, Norway; 5 Department of Psychology and Education Sciences, Transilvania University of Brasov, Brasov, Romania

**Keywords:** LGBT, discrimination, minority stress, internet, intervention, randomized controlled trial, Romanian teachers, Romania, school teacher, web-based, internet-based delivery, anxiety, empathy, self-efficacy, educators, online session, school, LGBTQ+

## Abstract

**Background:**

Discrimination can greatly impact both physical and mental health due to frequent stressors. Younger individuals, particularly those under the age of 17 years, are more adversely affected by victimization. Within the European Union, Romania exhibits poor rankings concerning LGBTQ+ (lesbian, gay, bisexual, transgender/transsexual, queer, and other minority sexual orientations and gender identities) inclusion, with large numbers of LGBTQ+ teenagers experiencing bullying due to their sexual orientation. Given that much of this discrimination and harassment occurs within schools, teachers and counselors are vital in affecting institutional change.

**Objective:**

This study aims to investigate the impact of an intervention on reducing prejudice against the LGBTQ+ community among Romanian teachers and counselors. Most prior interventions of this nature target Western, educated, industrialized, rich, and democratic populations.

**Methods:**

In this randomized controlled trial, we recruited 175 Romanian teachers via a national closed online user group and assigned them to either the experimental or control condition. Participants in the experimental condition received the intervention first and then completed the web-based outcome measures, while those in the control condition completed the measures first and then received the intervention. The intervention, designed for internet-based delivery, consisted of a 1-hour video session led by a pair of researchers. It blended educational information with testimonials of LGBTQ+ people, perspective-taking tasks, and a self-efficacy exercise. We measured LGBTQ+ prejudice (using Attitudes Toward Lesbians and Gay Men Scale, Homophobia Scale, and Attitudes Toward Homosexuals Scale), behavioral intentions, self-efficacy, perspective taking, intergroup disgust sensitivity, intergroup anxiety, empathy, factual knowledge about LGBTQ+ issues, as well as participants’ feelings toward lesbian, gay, and bisexual individuals.

**Results:**

Participants in the experimental group (n=89) showed significant reductions in prejudice when using the Attitudes Toward Lesbians and Gay Men Scale (*F*_1,173_=7.22; *P*=.008) when compared to the control group (n=86), but not when using the other 2 attitudinal scales. We also found that the experimental group had warmer feelings (*F*_1,173_=4.40; *P*=.04; *d*=0.32), were more likely to engage in supportive behaviors (*F*_1,173_=13.96; *P*<.001; *d*=0.56), displayed more self-efficacy (*F*_1,173_=9.14; *P*=.001; *d*=0.33), had more factual knowledge (*F*_1,173_=11.98; *P*=.001; *d*=0.52), and had a higher ability to take the LGBTQ+ perspective after controlling for contact (*F*_1,172_=4.77; *P*=.03; *d*=0.28). We did not observe significant differences in terms of intergroup disgust sensitivity (*F*_1,173_=0.816; *P*=.37), intergroup anxiety for either positive (*F*_1,173_=.383; *P*=.54) or negative emotions (*F*_1,173_=0.51; *P*=.48), or empathy (*F*_1,173_=0.02; *P*=.89).

**Conclusions:**

The intervention offers initial evidence for the effectiveness of a cost-effective and portable online resource for educators and high school counselors, particularly in regions where negative attitudes toward the LGBTQ+ community are prominent. The results show that integrating blended cognitive (information), affective (indirect contact and perspective taking), and behavioral (self-efficacy and empowerment) approaches is a promising avenue for intervention in producing positive outcomes related to LGBTQ+ issues within the school environment.

## Introduction

### Background

The LGBTQ+ (lesbian, gay, bisexual, transgender/transsexual, queer, and other minority sexual orientations and gender identities) community has historically faced great adversity, as centuries of prosecution have only recently begun to be overturned. The collapse of communism in Eastern Europe brought several changes in civil societies after they began shifting their focus to prioritizing human rights values and norms [1]. In Romania, homosexuality was officially decriminalized in 2001 because of external and international pressures [1]. Despite these changes, the consequences of the decades of oppression are seen even today, as attitudes toward LGBTQ+ individuals remain overall negative [2]. In fact, when it comes to LGBTQ+ inclusion, Romania tends to perform poorly compared to other EU countries. According to a recent survey [3,4], 45% of LGBTQ+ individuals in Romania reported experiencing discrimination in at least one aspect of their lives, but only 8% reported their experiences to an organization dealing with such issues. Additionally, 43% of the respondents said they faced harassment due to their sexual orientation or gender identity, the second-highest rate among all the EU countries.

The rates mentioned above are particularly concerning when viewed from the perspective of the minority stress model [5]. This model proposes that minorities face unique stressors because of stigmatization and discrimination, and that these can lead to detrimental effects on health. The stress caused by repeated experiences of discrimination can accumulate over time and negatively impact both physical and mental health in the long run.

Several studies have been conducted to test this model. In terms of physical health, a systematic review of 26 studies [6] found that minority stress impacted several physical health outcomes in sexual minorities, including cancer incidence, changes in cardiovascular function, and immune response. Additionally, a different systematic review [7] found that sexual minorities face significantly elevated risks for cardiovascular diseases. Similarly, mental health issues in LGBTQ+ individuals have been widely studied. A systematic review [8] revealed that sexual minorities had a higher risk for mental health issues, substance abuse, and suicide risk. In line with the minority stress model, evidence suggests that individuals who identify as LGBTQ+ also face several obstacles that negatively impact their mental health, such as discrimination, emotional distress, victimization, and barriers to accessing mental health services [9].

Moreover, although data suggest that minority stress affects all LGBTQ+ individuals, it seems that age impacts the stress felt by sexual minorities. Several studies have shown that younger individuals generally report more stress than older ones [10,11]. A meta-analysis revealed that victimization had more negative effects on LGBTQ+ individuals under the age of 17 years as compared to those aged more than 17 years [12]. These findings are corroborated by the impact on suicide risk. A meta-analysis of 35 studies [13] showed that suicide risk was higher for young LGBTQ+ individuals aged 12-20 years compared to cisgender straight young people. Furthermore, sexual minority youth are more likely to experience mental health issues such as anxiety, depression, and impaired academic performance [14]. This effect is especially prevalent within school environments, as supported by a systematic review showing significant negative effects of bias-based bullying for minorities, including LGBTQ+ students [15].

In fact, 44% of LGBTQ+ pupils aged 15 to 17 years in Romania have admitted to hiding their sexual orientation or gender identity in school, the third highest rate in the European Union after Croatia (51%) and Cyprus (47%), and significantly higher than in countries like the Netherlands (16%), France (20%), or Denmark (20%). Disturbingly, half of the respondents aged 15 to 17 years (50%) said that they were bullied at school because of their sexual orientation [3,4]. This finding is further supported by the United Nations Educational, Scientific and Cultural Organization report on school violence [16]. As such, given that discrimination and harassment against LGBTQ+ individuals are quite prevalent in Romanian schools and among students [3,4], it is therefore imperative to create programs that help reduce the stigma and prejudice that Romanian LGBTQ+ students face.

We decided to focus such efforts on teachers as they are perhaps the most significant group that can affect institutional change. Teachers can influence their students’ experiences by creating inclusive classroom norms, making these interventions most effective. In fact, survey evidence suggests that supportive communities and schools buffer the negative effects of bias-based bullying for LGBTQ+ students in schools [17] . This finding is backed up by qualitative data, with interviews in schools showing the importance of teacher attitudes and education in creating safe and inclusive environments for LGBTQ+ students [18]. Overall, a recent rapid realist review of interventions to promote LGBTQ+ inclusivity in schools [19] showed that interventions work best when the staff in the school are trained, including education on sexuality, gender issues, as well as how to be an effective ally. Teacher training was related to LGBTQ+ students experiencing less victimization, increased safety, greater self-esteem, improved mental health, and improved academic performance. Moreover, teacher training improved the effectiveness of teacher involvement in gay-straight alliances, which directly impacts LGBTQ+ youth’s outcomes in schools [20].

In this paper, we aim to present the findings of a teacher-oriented intervention whose protocol has already been published [21]. The main objective of the intervention is to use a 1-hour online session that focuses on education and contact as primary training elements. The session is meant to help high school teachers cope with cases of witnessing bullying acts against LGBTQ+ students and to improve their knowledge and attitudes toward LGBTQ+ students.

To create a safe and inclusive learning environment for LGBTQ+ students, it is crucial to not only address teacher biases toward them, but also to equip teachers with the skills and knowledge needed to intervene in LGBTQ+-related victimization. This includes providing teachers with training on how to recognize and respond to instances of LGBTQ+ bullying or discrimination, as well as model appropriate behaviors and attitudes toward LGBTQ+ students. By taking a proactive and comprehensive approach to support LGBTQ+ students, we can help ensure that they feel valued, respected, and included in the classroom, thus reducing the risk of significant mental and physical health costs.

According to a meta-analysis [22], interventions can be effective in reducing sexual prejudice. The effect size of these interventions can range from one-third to one-half of an SD. Furthermore, the analysis highlights the most effective strategies for reducing different outcomes. These strategies include educational interventions, contact with LGBTQ+ individuals, and a combination of education and contact. It is worth noting that the majority of interventions aimed at reducing homophobia have been conducted among undergraduate students in Western countries, with none conducted in Eastern Europe or other regions that still harbor clear animosity and prejudice toward the LGBTQ+ community. While some recent research has tested interventions in other countries such as Jamaica [23] or Brazil [24], in Romania (or Eastern Europe), no such interventions have been tested, particularly on teachers.

However, equally important is how the effect of these interventions is measured. The term “homophobia” has garnered a lot of attention since its inception in the 1970s [25]. Several researchers [26,27] have argued the term should not be used because of its inaccuracy, as the inclusion of the word “phobia” would suggest an anxiety-related measure rather than an attitudinal one. Additionally, the misnomer would mainly focus on attitudes toward gay men and leave out lesbians and bisexuals. This conceptual confusion has seeped into the measurements used when measuring the impact of the interventions. Currently, several measures exist that claim to test sexual prejudice, but few interventions have used more than 1 instrument. It would be, therefore, beneficial to include several measures to see which, if any, would better capture the effects of the intervention. Besides prejudice, behavioral, cognitive, and emotional outcomes are also vital in predicting and ensuring significant changes in the classroom.

In order to ensure the success of interventions, it is crucial to consider the cultural and institutional context in which they are implemented. A recent qualitative analysis [28] has shown that participants frequently criticize interventions for their lack of alignment with the context in which they are conducted. This can be used as a justification for resisting change. To achieve better outcomes, interventions should be tailored to the community’s unique characteristics, including their beliefs, values, traditions, and social norms.

### Aims and Hypotheses

The main objective of this research is to test an intervention plan that aims to improve Romanian teachers’ LGBTQ+ outcomes. Our intention is to consider particular cultural and institutional characteristics, rather than simply implementing previous intervention strategies. Our approach involves using education and contact as primary training elements, consistent with meta-analytic findings [22] on training effectiveness. However, we design the educational components according to the specific needs of our target group. For example, we incorporate information on the biological (rather than social) causes of homosexuality, as this is a common misbelief among Romanians. The protocol for this study is peer-reviewed and published in Research Protocols [21].

Additionally, we addressed potential feelings or perceptions of threat (such as the belief that exposure to LGBTQ+ individuals will “cause” children to become gay) as, according to intergroup threat theory [29], these are important predictors of anti-gay bias. We included several elements that had been found useful in other interventions, such as perspective-taking [30] and self-efficacy [31,32]. We also ensured that these elements were culturally appropriate. The trial protocol details were reported elsewhere [21], including a complete list of intervention components, subcomponents, and contents. Further details are given in Table 1 in a previous research paper [21].

Through a randomized controlled trial, we aim to measure the impact of the intervention on the attitudes, behavior, cognition, and emotions of teachers toward the LGBTQ+ individuals compared to those randomly assigned to the control condition. Additionally, we also measure the participants’ factual knowledge about LGBTQ+ issues in the classroom, as well as their attitudes toward LGBTQ+ individuals.

## Methods

### Ethical Considerations

The ethical review committee of the West University of Timisoara, Romania, approved this study (notice 74505/10.11.2022) after reviewing the procedure, measures, and materials used. Each participant was informed of the data collected and the details of the intervention on 3 separate occasions: upon signing up for the intervention session, upon beginning the intervention, and when responding to the questionnaires. Participants provided informed consent by clicking a radio button on an online sign-up page containing written information about the study.

Upon finishing the online multimedia intervention, respondents received an automatic unique code that they had to submit in a separate form to confirm their full participation in the intervention. Once participants confirmed their full participation, they were sent a certificate of participation that could be used to earn continuing education credits. They were also given the opportunity to enter a raffle to win a gift certificate worth 500 RON (equivalent to US $110 or €100).

All research data (outcome measures) were collected anonymously without recording any personal or identifiable information. However, to ensure that participants were indeed teachers, and to generate the participation certificates, the researchers collected personal information (email and name) that was stored in a separate database and never associated with the actual study results.

### Participant Recruitment and Eligibility Criteria

By working with the local center for educational resources and assistance to distribute an online message to all their national members, we aimed to recruit teachers or counselors employed in Romanian schools. The interested teachers and school counselors had the opportunity to sign up for 1 of the 17 advertised sessions across 3 months (from December 2022 to February 2023), scheduled at different times during the day, depending on their availability. To ensure more participation, the teachers who signed up for a session were sent a reminder email 24 hours before the commencement of the session.

All participants had to be either teachers or school counselors and be fluent in Romanian, as the intervention was conducted in this language. Additionally, because the trial was advertised and conducted online, respondents had to have basic computer or internet literacy to access the recruitment form and the Zoom meeting links (Zoom Communications, Inc).

### Study Design

The experimental design used in this intervention was a randomized controlled trial. We randomly assigned participants to either the experimental or control condition using a 2-group design. Those in the experimental condition received the intervention first and then completed the outcome measures, while those in the control condition completed the outcome measures first and then received the intervention. All participants had to fill out the outcome measures through the use of online questionnaires. This design was chosen for ethical reasons so that all participants would benefit from the intervention and associated resources by the end of their participation. Given the rigorous random assignment to the training and control conditions, we did not expect any baseline differences to influence the outcome measures. We did, however, conduct analyses with and without controlling for habitual contact with LGBT individuals. We chose this approach because discussions in the contact literature suggested that baseline levels of contact can potentially lead to a selection bias of participants to these studies [33,34] and as such, controlling those levels can help us isolate the intervention’s specific effect beyond habitual exposure.

Participants were grouped into sessions of up to 30 individuals, depending on their availability. Cluster randomization was done at the session level by the lead researcher, with each session being randomly assigned to either the experimental or control condition. The randomization was done with the use of an online number generator. Individual randomization within the session was not possible given that the intervention was presented to all participants at the same time.

Sessions were scheduled outside of typical working hours on different days and times to ensure there were no systematic biases due to participants’ session choice.

This study did not involve any risk to the participants’ physical or mental integrity, and no unintended harms were observed during implementation.

In addition to being published in an academic journal [21], the study protocol was also registered in an international clinical study registry (ISRCTN84290049) [35]. The study is reported in accordance with the CONSORT-EHEALTH (Consolidated Standards of Reporting Trials of Electronic and Mobile Health Applications and Online Telehealth) guidelines as per the completed checklist (Checklist 1).

### Intervention

To ensure a standardized experience for all participants, regardless of the session they attended, each session was led by a pair of researchers who were tasked with delivering scripted instructions, answering participant queries, and ensuring that the study was completed at a consistent pace with minimal distractions.

The stand-alone intervention was designed for internet-based delivery and was multimedia in nature. It contained a recorded animated presentation that contained information on terms, threat reduction, and effects of stigma, behavioral tools, testimonials of LGBTQ+ people, a perspective-taking task, and a self-efficacy exercise. In total, the intervention lasted 50 minutes. The full details on all the components of the intervention are presented in Table 1 in a previous research paper describing the protocol in detail [21].

### Sample Size Estimation

In order to determine the appropriate sample size for our study, we undertook a power analysis. Drawing on an average effect size of *d*=0.66 from a meta-analysis by Bartos et al [22], we aimed to achieve a statistical power of 0.80 as per the Cohen [36] recommendation that type II error should be limited to a probability of 0.20. Our analysis indicated a required sample size of 122 participants, although we elected to over-recruit in order to account for multiple outcomes. There was no pre-established stopping rule, and recruitment continued until all interested participants had been given the opportunity to participate in 1 of the 17 scheduled sessions.

All participants who completed their participation in the study were included in the data analysis, with no exclusions.

### Outcomes

#### Overview

In order to ensure the reliability of the final scores for each outcome, we verified that the Cronbach α coefficient passed the 0.7 threshold. Additionally, to ensure the accuracy of the results in our research, we used 3 different scales in measuring sexual prejudice and antigay bias.

#### Attitudes Toward Lesbians and Gay Men Scale: Measure of Sexual Orientation Prejudice

This 10-item scale measured beliefs and attitudes toward gay men and lesbians (“I think male homosexuals are disgusting” and “Sex between two women is just plain wrong.”) [37]. Items were rated on a Likert scale from 1 (strongly disagree) to 5 (strongly agree). After reverse coding 4 items, scores were averaged into a final score with higher values denoting more negative attitudes. Reliability for this measure was very good (Cronbach α=0.88).

#### Homophobia Scale (HS): Attitudes Toward Gay Individuals

This scale was composed of 25 items that assessed attitudes toward gay individuals, as well as social avoidance and aggression toward them [38]. Examples of items from the scale included, “Gay people make me nervous” and “I make derogatory remarks about gay people.” Participants rated each item on a Likert scale ranging from 1 (strongly disagree) to 5 (strongly agree). After reverse coding 9 items, the scores were averaged to obtain a final score. Higher scores indicate more negative attitudes toward gay people. The scale has shown excellent reliability with a Cronbach α of 0.93.

#### Attitudes Toward Homosexuals Scale: Assessment of Negative Beliefs

We used an additional scale to measure the attitudes toward and avoidance of gay people [39]. The 12 items included statements such as “Homosexuality is disgusting in the eyes of God,” and “If I can, I prefer to not be in the company of homosexuals.” Participants rated the statements on a Likert scale from 1 (strongly disagree) to 5 (strongly agree). Out of all, 5 items were reverse scored, and the statements were averaged into a final score with higher values denoting more negative attitudes. Reliability for this measure was excellent (Cronbach α=0.91).

#### Self-Efficacy

The original 10-item scale [40] was adapted for working with LGBTQ+ students in a school setting. The scale measured self-efficacy in dealing with issues related to LGBTQ+ students. Sample items included “If I try hard, I can solve difficult issues related to LGBTQ+ students” and “I can deal with unexpected situations that arise with LGBTQ+ students.” Ratings ranged from 1 (“Not at all true for me”) to 4 (“Perfectly true for me”), and higher scores indicating more self-efficacy in dealing with LGBTQ+–related behaviors in the classroom. The scale showed an excellent reliability with a Cronbach α of 0.92.

#### Behavioral Intentions

We used a 16-item scale to evaluate the propensity of teachers to exhibit supportive professional conduct in the classroom pertaining to LGBTQ+ issues [39,40], including discussing queries regarding sexual orientation with students or having books about gay and lesbian issues in the classroom. Each item was rated on a Likert scale ranging from 1 (strongly disagree) to 5 (strongly agree). A final score was obtained by averaging the responses, with higher scores indicating a greater willingness to engage in LGBTQ+ supportive behaviors in the classroom. The measure showed excellent reliability (Cronbach α=0.95).

#### Factual Knowledge About LGBTQ+ Issues

We designed a questionnaire consisting of 7 questions to evaluate the level of understanding of the participants on LGBTQ+ topics. The questions were carefully crafted based on the training content and covered a range of topics such as the biological basis of gender and the elevated risk of suicide among LGBTQ+ youth. The items were rated as either true or false, and the final score was calculated by adding up the number of correct responses. Participants with higher scores demonstrated a greater knowledge of and familiarity with LGBTQ+ issues.

#### Feeling Thermometer

We assessed the teachers’ attitudes toward gay, lesbian, and bisexual individuals using a feeling thermometer [41]. We asked them to rate their feelings toward each group using a sliding thermometer scale that ranged from 0 (indicating very negative feelings) to 100 (indicating very positive feelings). We then averaged the ratings for the 3 groups into an overall lesbian, gay, and bisexual (LGB) feelings thermometer. The scale showed excellent reliability (Cronbach α=0.98).

#### Intergroup Disgust Sensitivity

We used a 7-item scale to evaluate the repulsion toward LGBTQ+ groups [42]. The scale included statements such as “After shaking hands with someone who has a different sexual orientation, even if their hands were clean, I would want to wash my hands.” Participants rated items on a Likert scale ranging from 1 (strongly disagree) to 5 (strongly agree). One of the items was reverse-scored. The final score was calculated by averaging all the scores, with higher values indicating more disgust toward the LGBTQ+ community. Reliability for this measure was very good (Cronbach α=0.83).

#### Intergroup Anxiety

This scale consisted of 10 items that were designed to measure anxiety when interacting with people of another sexual orientation [43]. Participants were asked to rate the likelihood of feeling several emotions such as embarrassed, unsure, happy, or accepted, on a scale of 1 (not at all) to 10 (extremely). We calculated 2 scores, one for positive emotions and another for negative emotions. The reliability for both positive (Cronbach α=0.82) and negative emotions (Cronbach α=0.88) the reliability of the scale was very good.

#### Perspective Taking

This measure consisted of 5 items that assessed how well individuals can empathize with LGBTQ+ people [44]. Participants were asked to rate their ability to understand the issues that are important to LGBTQ+ people. The overall reliability of the scale was very good, with a Cronbach α score of 0.88.

#### Toronto Empathy Questionnaire

This scale consisted of 16 items that evaluated participants’ level of empathy toward others [45]. Examples of statements in the scale included “I feel upset when someone is treated disrespectfully.” Participants were asked to rate the statements on a Likert scale ranging from 1 (strongly disagree) to 5 (strongly agree). Among all, 7 items were reverse coded, and the final score was computed by averaging all items. Higher scores indicated higher levels of empathy. Reliability for this measure was very good (Cronbach α=0.80).

### Control Variables

#### Demographics

We asked respondents to indicate their age, gender, and sexual orientation. As all respondents were either teachers or counselors, they are required to have at least a bachelor’s degree in their respective field. Therefore, respondents were not asked to provide their education level.

#### Contact With LGBT People

We inquired about the frequency of contact (eg, speaking) with individuals who identify as gay, lesbian, bisexual, and transgender on a scale of 1 (almost daily) to 6 (never).

The Duke University Religion Index was used to measure religiosity [46]. The scale included 5 items that assessed religious involvement, organizational and nonorganizational religious activity, as well as subjective religiosity or intrinsic religiosity. The responses to each item were averaged to obtain a single score, which indicated the level of religiosity. Higher scores suggested greater religiosity. Cronbach α for this scale was 0.81, suggesting a very good reliability.

We also asked participants to indicate their political ideology on a 100-point scale from very conservative to very liberal or progressive.

### Statistical Analysis

Quantitative data from the surveys were analyzed using SPSS (version 24; IBM Corp) [47]. To examine our hypotheses, a series of one-way between-participants ANOVAs were performed. The objective of this analysis was to compare outcomes between the participants in the experimental and control groups. We also conducted the analyses while controlling for contact with LGBTQ+ individuals (ANCOVA), given that people’s different experiences may influence their response to training, particularly when it comes to affective outcomes. The results were presented as *F* test and *P* values, along with descriptive statistics such as n, mean, and SD. In order to estimate the effect size using means and SDs, we calculated Cohen *d*.

There was no deviation in the analysis from the registered protocol [21]. However, we decided to include a post hoc analysis in Multimedia Appendix 1, to explain some inconsistent results in the case of one outcome—the HS. We also controlled the analyses using the Benjamini-Hochberg false discovery rate (FDR) method to correct for any type I errors that could occur when testing for multiple hypotheses [48]. These analyses were conducted using the R programming language (version 4.5.1; R Foundation for Statistical Computing) [49]. The results are presented with both the corrected (noted as *P*_FDR_) and uncorrected *P* values.

## Results

### Descriptive Statistics

**Figure 1.**
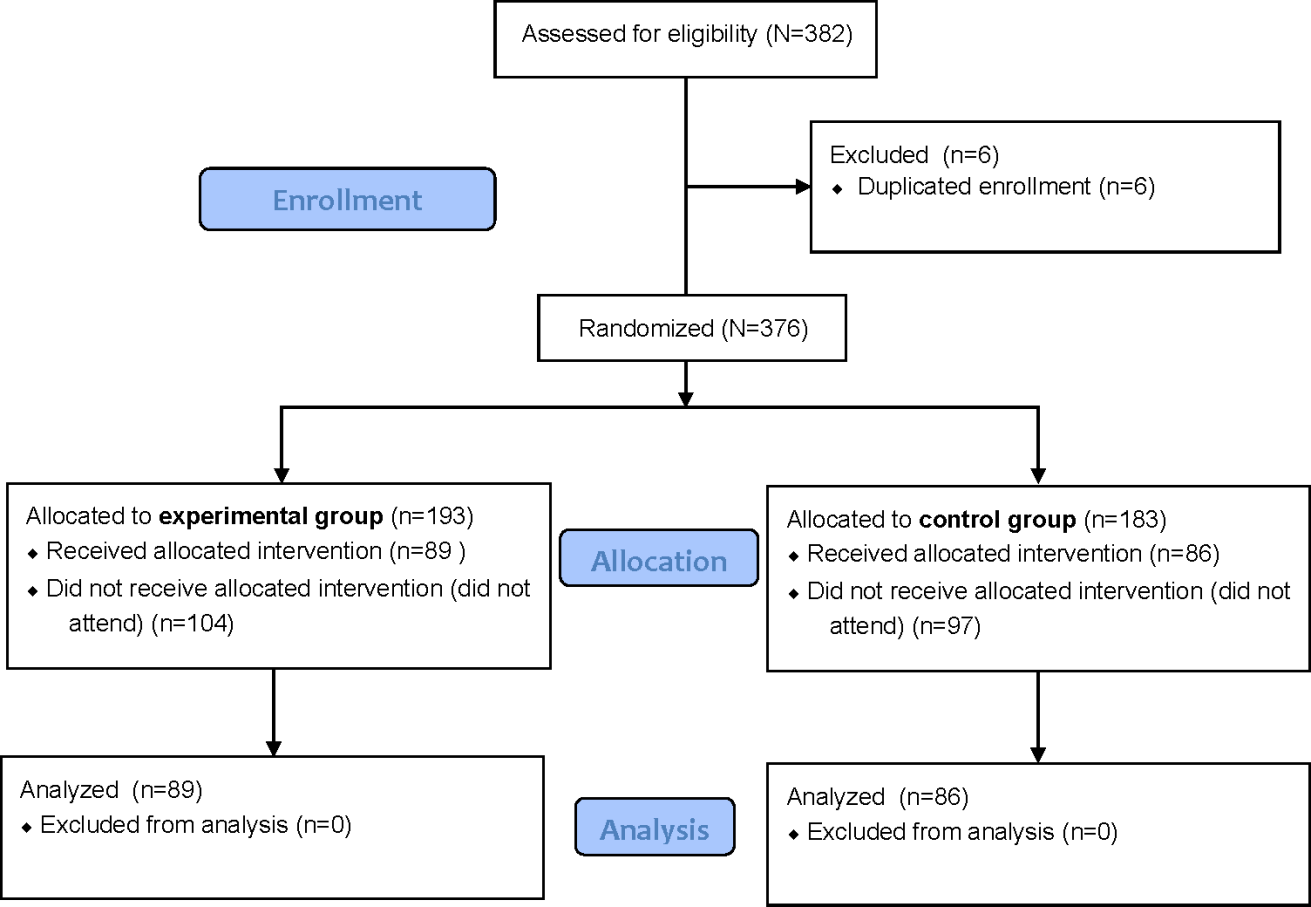
CONSORT (Consolidated Standards of Reporting Trials) flowchart.

A total of 382 individuals signed up to attend one of the advertised sessions. Further examination revealed that 6 of the submissions were duplicated, rendering 376 unique individuals that signed up. Of these, 175 out of 382 (45.81%) participants actually participated and finished the intervention, and 201 individuals did not attend despite repeated reminders. A CONSORT (Consolidated Standards of Reporting Trials) flowchart of the phases of the intervention is provided in Figure 1. A full summary of the descriptive statistics, for the full sample (N=175), as well as for the experimental group (n=89) and control group (n=86), is presented in Table 1. Also included in the table are the statistical analyses made to check whether there were any significant differences between the 2 groups. As can be seen, no significant differences were found between the experimental group and the control group in terms of age, gender, sexual orientation, religiosity, ideology, or LGBT contact. This confirms successful randomization across the 2 experimental conditions.

**Table 1.** Summary of descriptive statistics (N=175).

Variables	Full sample	Experimental group (n=89)	Control group (n=86)	Chi-square or *t* test (df)	*P* value
Age (y), mean (SD)	43.24 (7.77)	43.34 (7.97)	43.14 (7.62)	0.168 (173)^a^	.87
Gender, n (%)				1.65 (2)^b^	.44
Man	13 (7.4)	8 (9)	5 (5.8)		
Woman	161 (92)	81 (91)	80 (93)		
Nonbinary	1 (0.6)	0 (0)	1 (1.2)		
Sexual orientation, n (%)				4.06 (4)^b^	.40
Heterosexual	146 (83.4)	70 (78.7)	76 (88.4)		
Bisexual	5 (2.9)	3 (3.4)	2 (2.3)		
Other	6 (3.4)	4 (4.5)	2 (2.3)		
Do not know	2 (1.1)	2 (2.2)	0 (0)		
PNTS^c^	16 (9.1)	10 (11.2)	6 (7)		
Religiosity, mean (SD)	3.43 (0.98)	3.46 (0.94)	3.40 (1.03)	0.41 (173)^a^	.69
Ideology, mean (SD)	62.94 (24.86)	65.18 (23.9)	60.62 (25.75)	1.22 (173)^a^	.23
LGBT^d^ contact, mean (SD)	5.36 (1.03)	5.39 (0.99)	5.33 (1.08)	0.360 (173)^a^	.72

a 2-tailed* t* test (*df*).

b Chi-square (*df*).

c PNTS: prefer not to say.

d LGBT: lesbian, gay, bisexual, and transgender/transsexual.

### Outcomes

Significant differences were observed for the Attitudes Toward Lesbians and Gay Men Scale (*F*_1,173_=7.22; *P*=.008; *P*_FDR_=.02), where teachers in the control group had more negative attitudes (mean 2.70, SD 0.93) than those in the experimental group (mean 2.36, SD 0.77). The effect size, as indicated by Cohen *d*, was small to medium (*d*=0.41). Teachers who completed the intervention reported less negative attitudes toward lesbians and gays compared to teachers who did not complete the intervention. When controlling for the effect of contact, the experimental condition remained significant (*F*_1,172_=9.16; *P*=.003; *P*_FDR_=.009; *d*=0.46).

Interestingly, for the HS, while we did see lower scores for the experimental group (mean 1.95, SD 0.64) than the control group (mean 2.08, SD 0.76), these differences were not statistically significant (*F*_1,173_=1.58; *P*=.21; *P*_FDR_=.32). This effect remained nonsignificant when controlling for contact (*F*_1,172_=2.36; *P*=.13; *P*_FDR_=.20). The same can be observed for the Attitudes Toward Homosexuals Scale, where no statistically significant differences (*F*_1,173_=1.67; *P*=.20; *P*_FDR_=.32) were observed between the experimental group (mean 2.14, SD 0.65) and the control group (mean 2.31, SD 0.88). This effect also remained nonsignificant when controlling for contact (*F*_1,172_=2.40; *P*=.12; *P*_FDR_=.20).

Initially, significant differences were seen for the LGB feelings thermometer, in that respondents in the experimental group had higher (therefore warmer) feelings toward LGB community members (mean 71.80, SD 27.35) when compared to respondents in the control group (mean 62.64, SD 30.35*; F*_1,173_=4.40; *P*=.04; *P*_FDR_=.09; *d*=0.32), though the effect size was small, but after applying the LGB FDR correction, the main result failed to reach significance. When controlling for the effect of contact, the experimental condition remained significant even after applying the FDR correction (*F*_1,172_=5.82; *P*=.02; *P*_FDR_=.048).

We did not find significant differences between the 2 groups in terms of intergroup disgust sensitivity (*F*_1,173_=0.82; *P*=.37; *P*_FDR_=.49), intergroup anxiety for either positive (*F*_1,173_=0.38; *P*=.54; *P*_FDR_=.59) or negative emotions (*F*_1,173_=0.42; *P*=.52; *P*_FDR_=.59), or empathy (*F*_1,173_=0.02; *P*=.89; *P*_FDR_=.89). Even when controlling for the effects of contact with LGBTQ+ individuals, we did not see any significant results for intergroup disgust sensitivity (*F*_1,172_=1.11; *P*=.29; *P*_FDR_=.39), intergroup anxiety for either positive (*F*_1,172_=0.500; *P*=.48; *P*_FDR_=.52) or negative emotions (*F*_1,172_=0.51; *P*=.48; *P*_FDR_=.52), or empathy (*F*_1,172_=0.04; *P*=.84; *P*_FDR_=.84).

However, we did see significant differences in terms of behavioral intentions, in that respondents in the experimental group were more willing to engage in helping behaviors (mean 4.19, SD 0.68) than those in the control group (mean 3.75, SD 0.89; *F*_1,173_=13.96; *P<*.001; *P*_FDR_=.006; *d*=0.56), even after controlling for contact (*F*_1,172_=19.54; *P*<.001; *P*_FDR_=.004).

We also saw that factual knowledge was significantly higher in respondents from the experimental group (mean 5.04, SD 1.24) than those in the control group (mean 4.37, SD 1.32; *F*_1,173_=11.98; *P*=.001; *P*_FDR_=.006; *d*=0.52), even when controlling for contact (*F*_1,172_=12.03; *P*=.001; *P*_FDR_=.004). Furthermore, respondents in the experimental group (mean 3.18, SD 0.42) had significantly higher levels of self-efficacy than those in the control group (mean 2.94; SD 0.93; *F*_1,173_=9.14; *P*=.003; *P*_FDR_=.01; *d*=0.33), even after looking at the effects of contact (*F*_1,172_=11.82; *P*=.001; *P*_FDR_=.004).

Finally, the difference between the experimental and control conditions in terms of perspective taking was just shy of reaching significance (*F*_1,173_=3.27; *P*=.07; *P*_FDR_=.14; *d*=0.28), such that respondents in the experimental group had a slightly higher ability to take the LGBTQ+ perspective (mean 3.87, SD 0.69) than those in the control group (mean 3.66, SD 0.80). This analysis only reached significance after controlling for contact (*F*_1,172_=4.77; *P*=.03; *P*_FDR_*=.*06), although we note that the effect was small, and after applying the FDR correction, the results were again insignificant.

We conducted the above analyses in line with the preregistered research protocol published in a study by Latu et al [21].

## Discussion

### Principal Results

Our intervention suggests that even a 1-hour online session that combines informative materials (education, factual knowledge, and behavioral tools to address bullying and support LGBTQ+ students) and testimonials (vicarious contact) produces small to average positive effects on various LGBTQ+ outcomes. The intervention decreased teachers’ negative attitude toward LGBTQ+ topics, increased their self-efficacy in dealing with LGBTQ+ issues in the classroom, and led to a higher level of behavioral intentions to engage in supportive behaviors for LGBTQ+ students facing bullying in school contexts. It should be noted that the cognitive and behavioral outcomes were most stable, with the attitudinal outcome being relatively inconsistent across measures and types of analysis. Our inconsistent results across several scales of homophobia or antigay attitudes, together with our supplementary analyses, suggest that latent variables may be at play and that future research should systematically investigate the attitudinal components of homophobia or antigay bias for appropriate measurement.

The online intervention, however, did not lead to significant differences between the experimental and the control groups in terms of intergroup disgust or intergroup anxiety, nor on more general and nonspecific measures such as their empathy level toward other people. This may be because such emotional responses, compared to behavioral and cognitive components, are less malleable especially in the context of LGBTQ+ biases which are deeply ingrained in the Romanian mainstream culture. Our findings may also suggest that it was the educational and behavioral components of our intervention that were most effective. Improved affective outcomes, on the other hand, may result from the contact component of interventions [50]. Although vicarious contact was used in our intervention via recorded video testimonials, more direct and prolonged forms of contact with LGBTQ+ individuals may have the power to change affective responses.

The small to average effect sizes obtained for various outcomes align with an existing meta-analysis [22], in which interventions meant to reduce sexual prejudices obtained similar effect sizes, namely from one-third to one-half of an SD. As already explained elsewhere [21], most of these interventions have been implemented on undergraduate students from Western countries, and none has been implemented in Eastern Europe.

Likewise, our results suggest the opportunity to implement a multipurpose intervention, as suggested in prior studies [24]. Despite its shortness, the intervention showed multiple benefits at the attitudinal, cognitive, affective, and behavioral intention levels.

Taking together, our results are encouraging and a promising starting point for addressing LGBTQ+ biases in teachers. The findings show that an online intervention that is relatively low-cost in terms of personnel and resources could be implemented at a larger scale. Likewise, the session could represent a valuable resource to be added to a more complex intervention. The resource we developed could be an excellent tool for teachers in their professional development training when it comes to topics such as accepting sexual and gender diversity or when required to tackle LGBTQ+–related bullying in school contexts.

Another strength of this work is that the intervention was implemented in a country (Romania) which maintains strong negative attitudes toward LGBTQ+ individuals in the public sphere, with roots in conservative and religious beliefs. Such an educational resource turns away the attention from the moral or religious to psychological aspects such as perspective-taking and diversity acceptance, including vicarious contact with LGBTQ+ people and the problems they face in school settings. The emphasis on the humanistic view in teachers, backed up by education, seems to work even in less tolerant environments concerning LGBTQ+ issues. Therefore, the program behind the current intervention can be seen as a useful and more portable resource for guidance counselors in high schools as well as for other specialists who are interested in upscaling the positive results obtained in various high schools. When evaluating our findings, we believe that the intervention is especially useful given that it led teachers to perceive themselves as more equipped to intervene when LGBTQ+ students experience bullying acts.

### Limitations

A significant limitation of this study is the absence of long-term follow-up measurement of outcomes, with measurement being conducted in the same session as the intervention. Although these findings do not allow for inferences about long-term effects, we know that the outcomes of such interventions generally tend to be short-term. For example, a contact intervention [50] showed reduced LGBTQ+ negativity in the short term, but levels returned to baseline 7 days after. In contrast, those in the control condition who were not exposed to the intervention showed even more increased LGBTQ+ negativity 7 days later. Such findings suggest that our intervention, although short-term, can potentially lead to longer-term prevention of worsening of biases. We also suggest that, to secure long-term effects and change inclusivity norms in schools, our intervention could be part of a long-term curriculum of development for teachers.

Another limitation is that there may have occurred biases related to self-selection. Participants were included in the trial based on their voluntary consent and without any certain financial benefit (except for their inclusion in a raffle with a small chance of winning some financial benefits). Although their effort was minimal (attending an online 1-h session excluding the time required for the completion of the outcome questionnaires), it still could have led to the selection of a biased sample from the teachers’ population, as most likely teachers with strong negative attitudes toward LGBTQ+ people were more reluctant to attend our study. However, due to random assignment to conditions and the presence of significant effects, we believe there is sufficient evidence about the efficacy of the training.

Another limitation of the study is related to the lack of blinding to the experimental conditions. Because of the nature of the intervention and how it was organized, we could not effectively blind respondents and experimenters to the nature of how the trial worked, thus increasing the risk of a type I error. Future intervention could use artificial intelligence or other automated methods to deliver the intervention in a standardized way, to avoid any demand characteristics or experimenter effects.

A final issue is around the gender composition of our sample, which predominantly consists of women. This gender composition is in line with worldwide trends [51] but also national trends in the Romanian education system [52]. However, theoretically, future studies could investigate the effects of gender on the efficiency of such training tools and adapt them accordingly.

### Conclusions

This intervention is a promising resource that can serve as a valuable and relatively low-cost resource (no specialized human intervention is needed) for teachers and high school counselors, particularly in countries where negative attitudes toward LGBTQ+ people prevail. Our study also offers suggestions as to how such interventions could be improved and embedded in a longer-term program to increase classroom inclusiveness for LGBTQ+ students.

## Supplementary material

10.2196/63787 Multimedia Appendix 1 Additional analyses.

10.2196/63787 Checklist 1 CONSORT-EHEALTH (V 1.6.1) checklist.

## Abbreviations

CONSORT: Consolidated Standards of Reporting Trials
CONSORT-EHEALTH: Consolidated Standards of Reporting Trials of Electronic and Mobile Health Applications and Online Telehealth
FDR: false discovery rate
HS: Homophobia Scale
LGB: lesbian, gay, and bisexual
LGBTQ+: lesbian, gay, bisexual, transgender/transsexual, queer, and other minority sexual orientations and gender identities
