# Supplementary material for: An Internet-Delivered Intervention to Reduce LGBTQ+ Prejudice Among Romanian Teachers: Randomized Controlled Trial
Source: JMIR Hum Factors. 2026 Jan 16;13:e63787. doi: 10.2196/63787 (PMC12810945; doi:10.2196/63787)
Supplement: Multimedia Appendix 1 [file humanfactors-v13-e63787-s001.docx]

## Supplementary material

**Exploratory analyses**

Given the inconsistent results obtained for the three scales measuring attitudes towards homosexuality (ATLG, HS, ATH), and the well-known inconsistencies in measuring homophobia over time [1] we conducted additional analyses to better understand the effect of the intervention our attitudinal outcomes. To further explore this idea, an EFA with an orthogonal rotation (varimax) was conducted. The Kaiser-Meyer-Olkin Measure of Sampling Adequacy was .935, while Bartlett’s test of sphericity was statistically significant, with a p value of .000. Both measures indicate that the dataset is suitable for rotation [2,3].

The initial solution offered 7 factors that had an Eigenvalue greater than 1, which would explain a total of 65.99% of the variance. However, the Scree Plot suggested an inflection point after 4 factors. To settle this issue, we also conducted a Parallel Analysis using O’Connor’s syntax [4]. The results based on parallel analysis indicated that 4 factors should be considered [5,6]. As such, the EFA was conducted again, this time specifying the extraction of 4 total factors. These factors explain 58.83% of the total variance. After considering the items that are not cross-loaded, and based on the item texts, we have titled the four factors as *disgust* (composed of items 1,2, 6 and 7 of ATLG), *segregation* (items 10 and 11 of the HS), *negative behaviors* (items 6,9,13,14,15,17,19,24, and 25 of the HS and 4,7 and 11 of the ATH), and *acceptance* (or lack thereof) (items 3 and 8 of the ALTG). The one-way between-subjects ANOVAs were repeated for the newly created factors.

The results of the analysis showed that the *disgust* factor was significantly different (*F_1,173_*=9.21, *P*<.01; *d*=0.46) between the two groups, in that participants in the experimental group (M=2.33, SD=1.03) showed reduced disgust compared to participants in the control group (M=2.85, SD=1.23). When controlling for the effect of contact, the experimental condition still remained significant (*F_1,172_*=11.48, *P*=.001).

We also saw significant differences when looking at the *acceptance* factor, in that the experimental group (M=2.85, SD=1.16) had lower (therefore more positive) acceptance scores compared to the control group (M=3.29, SD=1.28), (*F_1,173_*=5.41, *P*<.05; *d*=0.36), though the effect size was small. When controlling for the effect of contact, the experimental condition still remained significant (*F_1,172_*=5.94, *P*<.05).

In terms of the *segregation* factor, while the means were slightly higher for the control group than the experimental group, the results were not statistically significant (*F_1,173_*=1.44, *P*=.23), even when controlling for the effect of contact (*F_1,172_*=2.10, *P*=.15).

Similar results were found for the *negative behaviors* factor, where no statistically significant differences were found (*F_1,173_*=.009, *P*=.923), even when controlling for contact effects (*F_1,172_*=.045, *P*=.83). This result may however be due to a floor effect, as the average score was 1.68 (SD= .62) for the control group and 1.67 (SD=.65) for the experimental group.

**Detailed analysis report for the EFA**

The initial solution offered 7 factors that had an Eigenvalue greater than 1, but the Scree Plot (Figure 1) suggests an inflection point after 4 factors. The Parallel Analysis using O’Connor’s syntax indicate 4 factors.


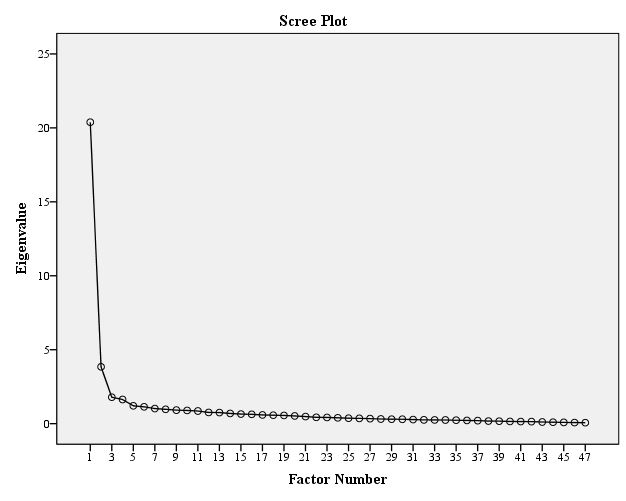


Figure S1. Scree Plot for the EFA

Table S1. Results of the Parallel Analysis

| Factor | Raw Data | Means | 95 Percentile |
| --- | --- | --- | --- |
| 1 | 20.10494 | 1.454166 | 1.592541 |
| 2 | 3.482076 | 1.323001 | 1.429578 |
| 3 | 1.5142 | 1.221497 | 1.311926 |
| 4 | 1.341594 | 1.134377 | 1.209505 |
| 5 | 0.84918 | 1.05919 | 1.131613 |

As such, the EFA was conducted again, this time specifying the extraction of 4 total factors. As can be seen in Table S2, these factors explain 58.83% of the total variance.

Table S2. Results of EFA – Total Variance Explained Factor

| **Factor** | **Initial Eigenvalues** | | | **Extraction Sums of Squared Loadings** | | | **Rotation Sums of Squared Loadings** | | |
| --- | --- | --- | --- | --- | --- | --- | --- | --- | --- |
|  | **Total** | **% of Variance** | **Cumul. %** | **Total** | **% of Variance** | **Cumul. %** | **Total** | **% of Variance** | **Cumul. %** |
| 1 | 20.385 | 43.372 | 43.372 | 19.974 | 42.497 | 42.497 | 8.021 | 17.066 | 17.066 |
| 2 | 3.844 | 8.178 | 51.550 | 3.345 | 7.117 | 49.614 | 7.400 | 15.745 | 32.811 |
| 3 | 1.789 | 3.805 | 55.355 | 1.402 | 2.982 | 52.596 | 6.821 | 14.512 | 47.323 |
| 4 | 1.633 | 3.475 | 58.830 | 1.231 | 2.620 | 55.216 | 3.710 | 7.893 | 55.216 |
| 5 | 1.205 | 2.564 | 61.394 |  |  |  |  |  |  |
| 6 | 1.142 | 2.429 | 63.823 |  |  |  |  |  |  |
| 7 | 1.023 | 2.176 | 65.999 |  |  |  |  |  |  |

Table S3 shows the factor loadings for the 4-factor solution. The bolded figures show the highest factor loadings; however, several items are cross-loaded (i.e. have loadings greater than .32 on more than one factor).

Table S3. Results of EFA – Factor loadings

| Scale | Item | Factor | | | |
| --- | --- | --- | --- | --- | --- |
|  |  | 1 - Disgust | 2 - Segregation | 3 - Negative Behavior | 4 - Acceptance |
| ATLG | ATLG1 | **0.758** | 0.197 | 0.071 | 0.233 |
|  | ATLG2 | **0.769** | 0.215 | 0.136 | 0.062 |
|  | ATLG3 | 0.122 | 0.129 | -0.013 | **0.656** |
|  | ATLG4 | **0.663** | 0.423 | 0.218 | 0.080 |
|  | ATLG5 | 0.387 | **0.464** | 0.349 | 0.450 |
|  | ATLG6 | **0.732** | 0.250 | 0.109 | 0.310 |
|  | ATLG7 | **0.782** | 0.315 | 0.129 | 0.174 |
|  | ATLG8 | 0.154 | 0.176 | 0.072 | **0.792** |
|  | ATLG9 | **0.704** | 0.341 | 0.227 | 0.113 |
|  | ATLG10 | 0.388 | 0.350 | 0.342 | **0.457** |
| HS | HS1 | **0.466** | 0.442 | 0.341 | 0.028 |
|  | HS2 | **0.504** | 0.201 | 0.377 | 0.226 |
|  | HS3 | 0.449 | 0.424 | 0.285 | **0.458** |
|  | HS4 | 0.322 | **0.546** | 0.395 | 0.083 |
|  | HS5 | 0.406 | **0.540** | 0.222 | 0.138 |
|  | HS6 | 0.263 | 0.182 | **0.563** | -0.057 |
|  | HS7 | 0.333 | **0.566** | 0.156 | 0.263 |
|  | HS8 | **0.605** | 0.459 | 0.121 | 0.432 |
|  | HS9 | 0.112 | 0.175 | **0.624** | 0.018 |
|  | HS10 | 0.221 | **0.751** | 0.200 | 0.205 |
|  | HS11 | 0.221 | **0.744** | 0.247 | 0.227 |
|  | HS12 | **0.464** | 0.307 | 0.187 | 0.427 |
|  | HS13 | 0.049 | 0.068 | **0.678** | 0.017 |
|  | HS14 | 0.188 | 0.216 | **0.543** | 0.038 |
|  | HS15 | 0.169 | -0.023 | **0.708** | 0.046 |
|  | HS16 | 0.414 | 0.375 | 0.259 | **0.500** |
|  | HS17 | -0.147 | 0.076 | **0.609** | 0.049 |
|  | HS18 | 0.406 | **0.595** | 0.232 | 0.228 |
|  | HS19 | 0.178 | 0.181 | **0.598** | 0.103 |
|  | HS20 | 0.303 | **0.467** | 0.247 | 0.227 |
|  | HS21 | 0.394 | 0.477 | **0.497** | 0.036 |
|  | HS22 | **0.401** | 0.356 | 0.202 | 0.131 |
|  | HS23 | 0.386 | 0.249 | **0.422** | 0.204 |
|  | HS24 | 0.013 | 0.046 | **0.517** | 0.066 |
|  | HS25 | 0.175 | 0.219 | **0.621** | 0.139 |
| ATH | ATH1 | 0.357 | **0.472** | 0.404 | 0.052 |
|  | ATH2 | 0.377 | **0.502** | 0.069 | 0.224 |
|  | ATH3 | **0.526** | 0.464 | 0.161 | 0.489 |
|  | ATH4 | 0.269 | 0.248 | **0.640** | 0.141 |
|  | ATH5 | 0.322 | **0.711** | 0.169 | 0.219 |
|  | ATH6 | 0.247 | **0.546** | 0.367 | 0.185 |
|  | ATH7 | 0.271 | 0.251 | **0.586** | 0.192 |
|  | ATH8 | 0.284 | **0.555** | 0.416 | 0.135 |
|  | ATH9 | **0.525** | 0.434 | 0.166 | 0.289 |
|  | ATH10 | 0.405 | 0.255 | **0.504** | 0.178 |
|  | ATH11 | -0.001 | 0.246 | **0.379** | 0.064 |
|  | ATH12 | **0.444** | 0.391 | 0.097 | 0.438 |
| Extraction Method: Principal Axis Factoring.   Rotation Method: Varimax with Kaiser Normalization. | | | | | |
| a. Rotation converged in 6 iterations. | | | | | |

**References**

1. Costa AB, Bandeira DR, Nardi HC. Systematic review of instruments measuring homophobia and related constructs. J Applied Social Pyschol. 2013;43(6):1324-1332.
2. Kaiser HF, Rice J. Little jiffy, mark IV. Educational and psychological measurement. 1974 Apr;34(1):111-7. <https://doi.org/10.1177/001316447403400115>
3. Bartlett MS. Tests of significance in factor analysis. British journal of Psychology. 1950; 3:77-85.
4. O’connor BP. SPSS and SAS programs for determining the number of components using parallel analysis and Velicer’s MAP test. Behavior research methods, instruments, & computers. 2000 Sep;32(3):396-402. <https://doi.org/10.3758/BF03200807>
5. Hayton JC, Allen DG, Scarpello V. Factor retention decisions in exploratory factor analysis: A tutorial on parallel analysis. Organizational research methods. 2004 Apr;7(2):191-205. <https://doi.org/10.1177/1094428104263675>
6. Tabachnick BG. & Fidell, LS (2001). Using multivariate statistics. Bost. Allyn Bacon. Bamberger. 7;31.
